# Bacteriological quality of fresh and processed black soldier fly *Hermetia illucens* larvae reared on chicken manure in Kitwe, Zambia

**DOI:** 10.1128/spectrum.00570-24

**Published:** 2025-06-20

**Authors:** Prudence Mapiki, Ester Laiser, Joyce Mufungwe, Misheck Shawa, Mazuba Siamujompa, Todd Johnson, Ngawo Namukonde, Phenny Mwaanga, Bernard Mudenda Hang'ombe

**Affiliations:** 1 Department of Para-Clinical Studies, University of Zambia 108234, Lusaka, Zambia; 2 Department of Animal Sciences, University of Zambia 108234, Lusaka, Zambia; 3 Department of Zoology and Aquatic Sciences, Copperbelt University 108291, Kitwe, Zambia; 4 Department of Biological Sciences, Copperbelt University 108291, Kitwe, Zambia; 5 Department of Environmental Engineering, Copperbelt University 108291, Kitwe, Zambia; University of Mississippi, University, Mississippi, USA

**Keywords:** black soldier fly, bacteria, chicken manure, antimicrobial resistance

## Abstract

Black soldier fly larvae (BSFL) have high nutrient content and are rapidly becoming an alternative protein source for animal feed. However, microbial contamination is a potential risk due to the environment in which they are reared. This study assessed the bacteriological quality of fresh and processed BSFL by comparing the processed BSFL using two traditional methods (oven-dried and sun-dried), on their effectiveness at reducing the bacterial load and further elucidated bacterial composition. PCR was used to identify extended-spectrum β-lactamase (ESBL) and *mec*A genes in *Escherichia coli* and *Staphylococcus* spp., respectively. A total of 51 fresh BSFL samples were collected from a commercial poultry farm in Kitwe, Zambia. The results showed various bacterial genera, with a higher diversity among gram-positive isolates. The comparison of the effectiveness of two traditional processing methods for BSFL, sun-drying and oven-drying, showed that both methods significantly reduced the bacterial load, with oven-drying causing a larger reduction. While various genera were identified, we focused on *E. coli* and *Staphylococcus* spp. This is because some *E. coli* harbor ESBLs that hydrolyze β-lactam antibiotics like cephalosporins and penicillin, leading to resistance. Similarly, the genus *Staphylococcus* was selected since some strains are potentially pathogenic and contain the *mec*A gene that encodes resistance to β-lactam antibiotics. Molecular characterization of the isolated strains revealed *bla*_CTX-M_ and *bla*_TEM_ genes among *E. coli*, but the *mecA* gene was not detected among *Staphylococcus*. This study revealed that BSFL harbor bacteria of zoonotic significance, emphasizing the need for good processing methods to eliminate potential risks.

**IMPORTANCE:**

Isolation and identification of *Escherichia coli* and *Staphylococcus* spp. in processed black soldier fly larvae (BSFL) samples meant for animal feed indicate insufficient processing methods and pose a public health risk. For instance, some *E. coli* harbor extended-spectrum β-lactamases (ESBLs) that hydrolyze β-lactam antibiotics like cephalosporins and penicillin, leading to resistance. In addition, some *E. coli* commensals can transfer antimicrobial resistance genes to pathogenic bacteria through horizontal gene transfer using various mobile genetic elements, leading to resistance. Similarly, for *Staphylococcus* spp., some strains of the genus *Staphylococcus* are potentially pathogenic and contain the *mec*A gene that encodes resistance to β-lactam antibiotics. In this study, we used PCR to screen *E. coli* isolates for the two commonly reported ESBL genes in Zambia, *bla*_CTX-M_ and *bla*_TEM_, and Sanger sequencing was used to reveal *bla*_CTX-M_ gene alleles. Our results highlight the importance of using adequate processing methods for BSFL to eliminate potential health risks to animal feed.

## INTRODUCTION

The black soldier fly (BSF) is an insect that inhabits tropical and warm-temperate regions worldwide. It is known to feed on a wide range of biological wastes in its larval stages (1). Therefore, the BSFs are ideal for industrial applications as they grow rapidly, have high nutritional value, and typically do not harbor pathogenic microbes (2). Furthermore, the BSF larvae (BSFL) can be reared in organic waste, converting low-quality materials into valuable biomass (3). Lastly, the production of BSFL is simple, cost-effective, and environmentally sustainable (4, 5). These favorable properties of BSFL could be used for human and animal welfare. For example, BSFL proteins can potentially replace soy and fish meal, commonly used for animal feed (4), indirectly reducing deforestation from clearing land for soy production, where human welfare is enhanced. In addition, BSFL lipids may replace palm kernel oil and contribute to the conservation of tropical forests (4). Other benefits include using BSFL-based lipids for biodiesel production and as bio-chemicals for cosmetics (4, 6). However, due to the rearing environment, pathogens in the substrate may be transferred to the larval intestinal tract and colonize or infect the animals fed with BSFL-based feed or people consuming the animal products (7). For example, *Salmonella* spp. and *Escherichia coli* found in BSFL-rearing substrates have been associated with zoonotic diseases in human consumers (8). Thus, utilizing edible insects such as BSFL on a commercial scale requires suitable processing methods or techniques that ensure quality and safety improvement (9).

Many traditional processing methods, such as boiling, roasting, sun-drying, oven-drying, toasting, frying, smoking, or a combination of these, have been used to process insects in Africa (10). However, little is known about the effectiveness of these methods when the insects are directly harvested from the wild. There is also a scarcity of literature on assessing the presence of antimicrobial resistance (AMR) in bacteria found in the larvae from diverse substrates in natural environments. The available literature on the processing of insects is from controlled environments, with little data on AMR (11). Since habitat heterogeneity can lead to differences in insect quality and safety (10), assessing localities where the mass rearing of BSFL is under consideration becomes vital.

This study aimed to determine the effectiveness of commonly used processing methods in improving the hygienic quality of wild BSFL obtained from a poultry farm’s waste dumpsite in Zambia’s Kitwe district. BSFL were collected from a poultry farm’s waste dumpsite because the larvae naturally thrive in organic waste environments, such as chicken manure, and farmers often rear the larvae on similar waste substrates and later use the larvae as animal feed. Furthermore, the study also aimed to characterize BSFL-associated bacteria in terms of AMR phenotypically and genotypically when reared in such environments.

## MATERIALS AND METHODS

**Fig 1:**
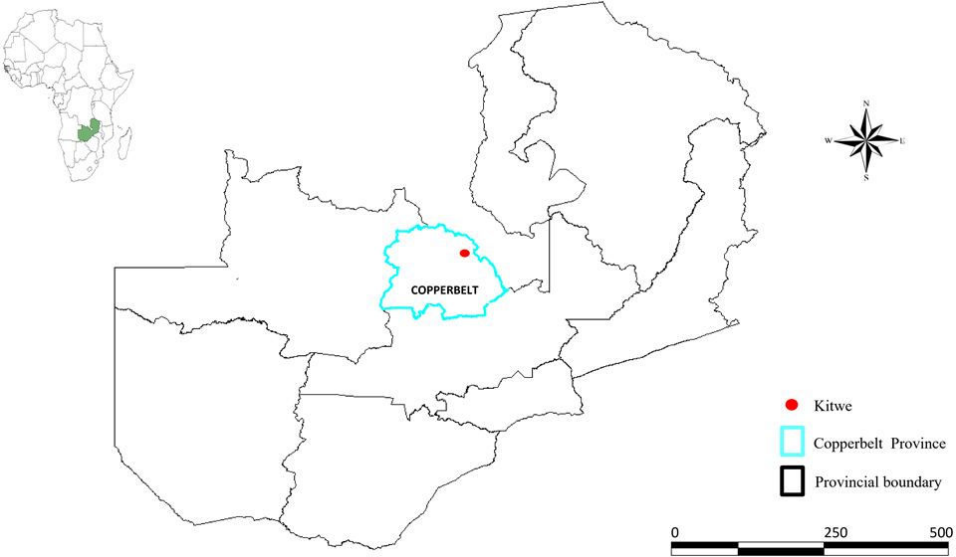
Map of Zambia showing the location of Kitwe town.

BSFL were collected at a poultry farm’s waste dumpsite on the outskirts of Kitwe town, located in the Copperbelt Province of Zambia (Fig. 1). Despite Kitwe having several poultry dumpsites, samples were purposely collected from the largest chicken manure-producing sites in Kitwe. The farm uses an intensive rearing method where chickens are housed in cages with automated drinkers, heating, and feed provisions. In this case, the dumpsite is where poultry carcasses, feed wastes, excrements, process wastewater, and other wastes associated with poultry confinement from a poultry feeding operation are disposed of. Thus, we collected BSFL from this dumpsite as part of the Copperbelt University (CBU) research project to determine the sustainable production of BSFL as animal feed in Zambia. The dumpsite covers an area of approximately 10,000 m^2^.

The BSFL samples collected from the dumpsite were exposed to two different processing conditions, oven-drying and sun-drying, with non-dried samples (fresh samples) taken as a control. Three sets of 17 BSFL samples were collected simultaneously from different heaps selected randomly from the dumpsite. Each BSFL sample set had an average weight of 250 g, with actual weights ranging from 240 g to 260 g. The larvae were collected using forceps while wearing gloves and placed in small, clean buckets. After collection, the samples were transferred to the laboratory at the CBU and washed with running tap water using a sieve (1 mm mesh size) for 1 minute to remove residues from the surface of the larvae. Then, the samples were kept in clean zip lock bags placed on icepacks, transported to the University of Zambia (UNZA), School of Veterinary Medicine, and frozen at −20°C within 24 hours of collection, before further processing

### Processing

Upon arrival at the laboratory, each sample was assigned a unique identification number before processing. Samples were then dried according to the study’s selected methods (sun-drying and oven-drying) before being processed for bacterial load count and identification.

### Sun-drying

Freshly thawed BSFL weighing 100 g were first disinfected with 70% ethanol and placed on sterile plastic plates in the sun. The BSFL were disinfected with 70% ethanol to eliminate any surface and external microbial contamination, and thus limit the study to gut microbiota. Then, the larvae were dried according to the conditions of the day temperatures. Samples were considered dry when the net weight of the BSFL was within 25%–35% of the original fresh larvae. The samples were removed from the sun, and microbiological analyses were conducted.

### Oven-drying

Freshly thawed BSFL weighing 100 g were first disinfected with 70% ethanol and put on kitchen paper towels in trays with holes for air circulation. The larvae were then dried at 65°C in a one-night phase of 16 hours, followed by a two-day phase of 4 hours each, where trays were reshuffled in between phases accordingly. The process was completed when the larvae had a dry texture and shrunken shape, weighing 25%–35% of the original fresh larvae. The samples were then subjected to microbiological analysis.

### Fresh sample of BSFL

Freshly thawed BSFL weighing 100 g were first disinfected with 70% ethanol and then rinsed twice with distilled water. One gram of the fresh larvae was weighed, homogenized, and used for bacterial enumeration.

### Enumeration, isolation, and identification of bacteria

One gram of homogenized larvae from all three BSFL sample groups (fresh, oven-dried, and sun-dried) was transferred into individual test tubes containing 9 mL of normal saline. Ten-fold serial dilutions were carried out, and 100 µL was aseptically surface-plated on nutrient agar (HI Media M001, HiMedia Laboratories, India). After incubation at 37°C for 24 hours, the bacterial colonies were counted, and the number of colony-forming units per mL (CFU/mL) was calculated. MacConkey agar (HiMedia Laboratories, India) was then used to isolate and differentiate non-fastidious gram-negative bacteria, while blood agar (HiMedia Laboratories, India) was applied for fastidious bacteria (particularly *Staphylococcus*). Morphologically distinct colonies were picked and streaked on new culture media (MacConkey and blood agar), then incubated at 37°C for 24 hours to obtain pure cultures. Gram staining technique, catalase test, mannitol salt agar (MSA), and eosin methylene blue (EMB) were used to characterize the bacteria. Colonies obtained from blood agar were inoculated on MSA, which is selective for the *Staphylococcus* genus. Similarly, colonies from MacConkey were inoculated on EMB, which is selective for gram-negative bacteria.

### Antimicrobial susceptibility tests

The AMR profiles of *Staphylococcus* spp. and *E. coli* isolates were determined using the antibiotic disk diffusion on Mueller-Hinton agar (MHA) from Thermo Fisher Scientific. First, the pure cultures were aseptically applied to the MHA plates using sterile cotton swabs. Then, the antibiotic disks were gently placed on the inoculated MHA plate using sterile forceps and incubated at 37°C for 24 hours. Antibiotics were selected based on the common antibiotics used in hospitals and recommended by the World Health Organization. A total of 10 commonly used antibiotics in humans belonging to six classes, namely cephalosporins (cefepime [FEP, 30 µg]), cefotaxime (CTX, 30 µg), quinolones (ciprofloxacin [CIP, 5 µg]), nalidixic acid (NAL, 30 µg), aminoglycosides (gentamicin [GEN, 10 µg]), penicillin (ampicillin [AMP, 10 µg]), sulfonamides (co-trimoxazole [SXT, 25 µg]), and phenols (chloramphenicol [CHL, 30 µg]), streptomycin (STR, 10 µg), tetracycline (TET, 30 µg) were used. The zone of inhibition (diameter) of each antibiotic disk was measured in millimeters (mm) using a ruler, and the results were defined according to the Clinical Laboratory Standards Institute (12) and the European Committee for Antimicrobial Susceptibility Testing (13). The susceptibility of a given bacterium to an antibiotic was determined based on the breakpoints of both CLSI and EUCAST guidelines. Isolates that showed resistance to three or more classes of antimicrobials were considered multidrug-resistant (MDR), as proposed by Magiorakos et al. (14).

### Screening for extended-spectrum β-lactamase and *mecA* genes

The isolated *E. coli* were streaked on MacConkey agar, supplemented with 1 µg/mL CTX, while catalase-positive *Staphylococcus* spp. were inoculated on lysogeny broth containing 64 µg/mL of cloxacillin. This was followed by incubation at 37°C for 18 hours. The colonies obtained were subjected to genomic DNA (gDNA) extraction using a QIAamp DNA Kit (Qiagen, Hilden, Germany) according to the manufacturer’s instructions. The gDNA was then subjected to PCR using TaKaRa Ex Premier DNA Polymerase under specified conditions. The primers used are listed in Table 1. The obtained PCR amplicons were subjected to the Wizard SV Gel and PCR Clean-Up System (Promega, USA), and then sequencing PCR was done with the Big Dye Terminator Cycle by Sanger sequencing using the SeqStudio Genetic Analyzer (Applied Biosystems, USA). Finally, the obtained sequences were edited and assembled with SnapGene software, followed by a BLAST search in GenBank.

**TABLE 1:** Primers used in this study

Name	Sequence	Size (bp)	Target	Reference
CTX-MA1	*SCSATGTGCAG≠YACCAGTAA	544	*bla*_CTX-M_	(15)
CTX-MA2	CCGC¥RATATGRTTGGTGGTG			
yaiO-F	TGATTTCCGTGCGTCTGAATG	115	*E. coli*	(16)
yaiO-R	ATGCTGCCGTAGCGTGTTTC			
SHV-F2	AGGATTGACTGCCTTTTTG	392	*bla*_SHV_	(17)
SHV-R2	ATTTGCTGATTTCGCTCG			
TEM-C	ATCAGCAATAAACCAGC	516	*bla*_TEM_	(18)
TEM-H	CCCCGAAGAACGTTTTC			
OXA-F	ATATCTCTACTGTTGCATCTCC	619	*bla*_OXA_	(17)
OXA-R	AAACCCTTCAAACCATCC			
MRSA₁	AAAATCGATGGTAAAGGTTGGC	533	*mec*A	(19)
MRSA₂	GTTCTGCAGTACCGGATTTGC			

### Data processing and analysis

Data on bacterial loads were cleaned using pivot tables in Microsoft Excel, then imported to Jamovi 2.3.21.0 for a one-way analysis of variance (ANOVA) test to check for significant mean differences among the treatment groups. Data cleaning in this study means removing duplicates and organizing the data in a format suitable for analysis. Data on antimicrobial susceptibility test (AST) were recorded in Microsoft Excel 2007 and imported into R for manipulation and visualization using dplyr v1.0.7 (20) and ggplot2 v3.3.5 (21).

## RESULTS

### Number of isolates

The result revealed that fresh BSFL had more isolates, 79 (60%), followed by sun-drying with 37 (28%), and lastly oven-drying with 15 (12%), as shown in Table 1. *E. coli* isolates were highest in the fresh samples, 19 (24%), followed by 9 (19%) in the sun-dried samples, and least 2 (13%) in the oven-dried samples. *Staphylococcus* isolates were 28 (35%) in the fresh samples, 17 (46%) in sun-dried samples, and 5 (33%) in oven-dried samples. In the fresh samples, several other bacterial isolates (*Bacillus, Clostridium*, *Streptococcus,* and sporulated *Bacillus)* were identified, while in the sun-dried and oven-dried samples, only *Bacillus* was identified (Table 2).

**TABLE 2:** Numbers of isolates in fresh, sun-dried, and oven BSFL samples

Identified bacterial genera	No. of isolates		
	Fresh samples	Sun-dried samples	Oven-dried samples
*Bacillus*	20	11	8
*Clostridium*	1	–	–
*Escherichia*	19	9	2
*Proteus*	1	–	–
*Staphylococcus*	28	17	5
*Streptococcus*	8	–	–
Sporulated *Bacillus*	2	–	–
Total	79	37	15

### Bacterial load

To measure the efficiency of various traditional drying methods, we compared the CFU from samples subjected to oven-drying and sun-drying, with fresh samples acting as controls, grown on nutrient agar. We noted a statistically significant difference. Specifically, the results revealed that the average total plate count on homogenized BSFL was 2.9 × 10^12^ CFU/mL, 3.2 × 10^3^ CFU/mL, and 2.2 × 10^4^ CFU/mL for fresh, oven-dried, and sun-dried methods, respectively. The analysis of the colony count resulted in a significant difference (*P* < 0.001) in the averages of colonies isolated from the three different groups, as indicated in Tables 3 and 4.

**TABLE 3:** Sample comparison using one-way ANOVA and treatment means

	*F*	df1	df2	*P*
Colony count				
Welch’s	38.7	2	23.8	<0.001
Fisher’s	24.9	2	48	<0.001

**TABLE 4:** Sample group descriptives

Treatment	*N*	Mean	SE	SE
Colony count				
Fresh	17	2.90E+07	2.08E+07	5.04E+06
Oven-dried	17	3,229	3,679	892
Sun-dried	17	22,471	10,075	2,444

### AST for *E. coli*

*E. coli* isolates from fresh, sun-dried, and oven-dried samples, grown on MacConkey agar, were subjected to AMR tests using five antibiotics: ampicillin (AMP), chloramphenicol (CHL), ciprofloxacin (CIP), cefotaxime (CTX), and gentamicin (GEN) using both EUCAST and CLSI standard guidelines. We observed that, in the fresh samples, *E. coli* resistance to AMP and CIP was similar at 26.3% and 63.2%, respectively, while resistance to CHL (31.6%) and GEN (68.4%) was higher for EUCAST compared to CHL (21.1%) and GEN (47.4%) under CLSI guidelines. Furthermore, *E. coli* resistance to CTX was higher (84.2%) under CLSI compared to 63.2% under EUCAST.

**Fig 2:**
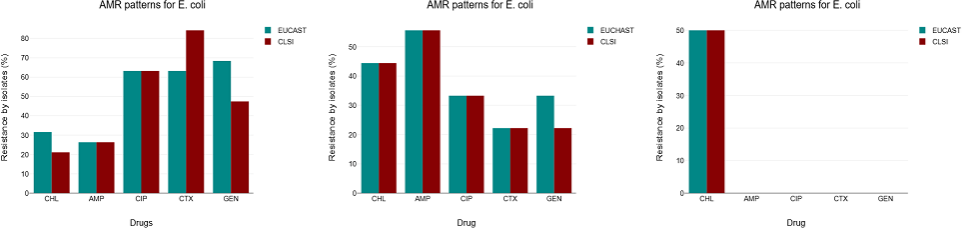
*E. coli* resistance to common antibiotics using EUCAST and CLSI protocols. AMP, ampicillin; CHL, chloramphenicol; CIP, ciprofloxacin; CTX, cefotaxime; GEN, gentamicin.

In the sun-dried samples, four of the five antibiotics had similar resistance patterns under the two protocols (AMP, 55.6%; CHL, 44.4%; CIP, 33.3%; and CTX, 22.2%) while resistance to GEN was higher (33.3%) under EUCAST than CLSI (22.2%). In addition, oven-dried samples showed the same *E. coli* resistance to CHL, which was 50% under both protocols, while there was 0% for other antibiotics (Fig. 2).

### AMR patterns in *Staphylococcus* species

**Fig 3:**
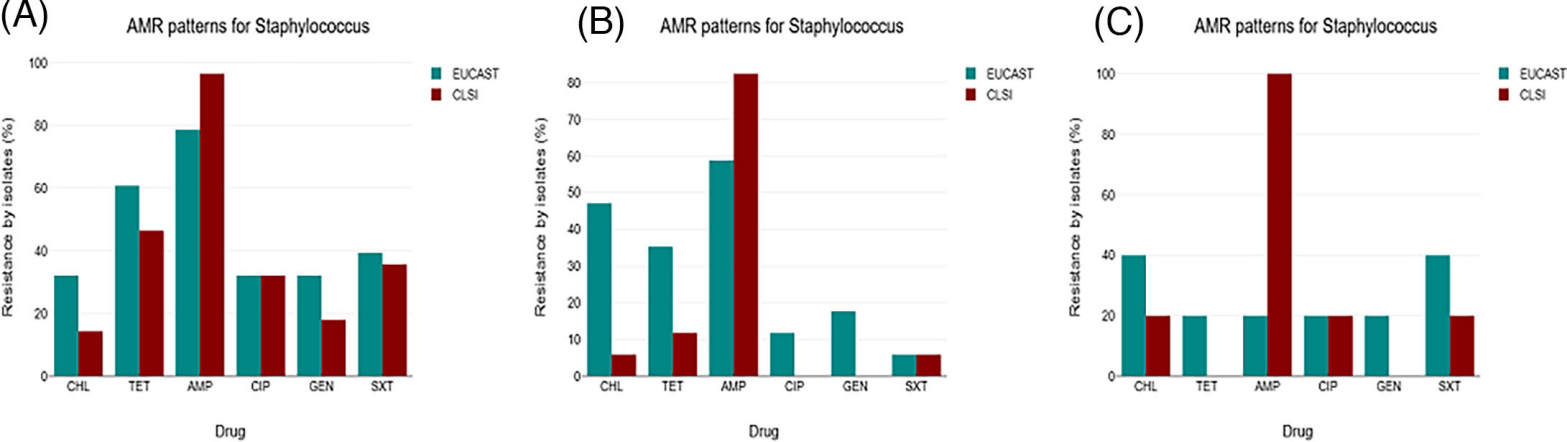
Resistance patterns of *Staphylococcus* against commonly used antibiotics: (A) fresh samples, (B) oven-dried samples, and (C) sun-dried samples. AMP, ampicillin; CHL, chloramphenicol; CIP, ciprofloxacin; GEN, gentamicin; TET, tetracycline; sxt, co-trimoxazole.

Isolates *of Staphylococcus* spp. from fresh, sun-dried, and oven-dried samples grown on blood agar were subjected to AMR tests using six antibiotics: ampicillin, chloramphenicol, ciprofloxacin, gentamicin, tetracycline (TET), and sulfamethoxazole/trimethoprim (SXT). The results showed differences in resistance rates between the two standards. Specifically, resistance rates to AMP were higher (>80%) under the CLSI protocol, regardless of the processing method. However, for other antibiotics, the EUCAST protocol exhibited resistance rates higher than or equal to the ones displayed by the CLSI guidelines (Fig. 3).

### Multi-drug resistance in *E. coli* isolates

**Fig 4:**
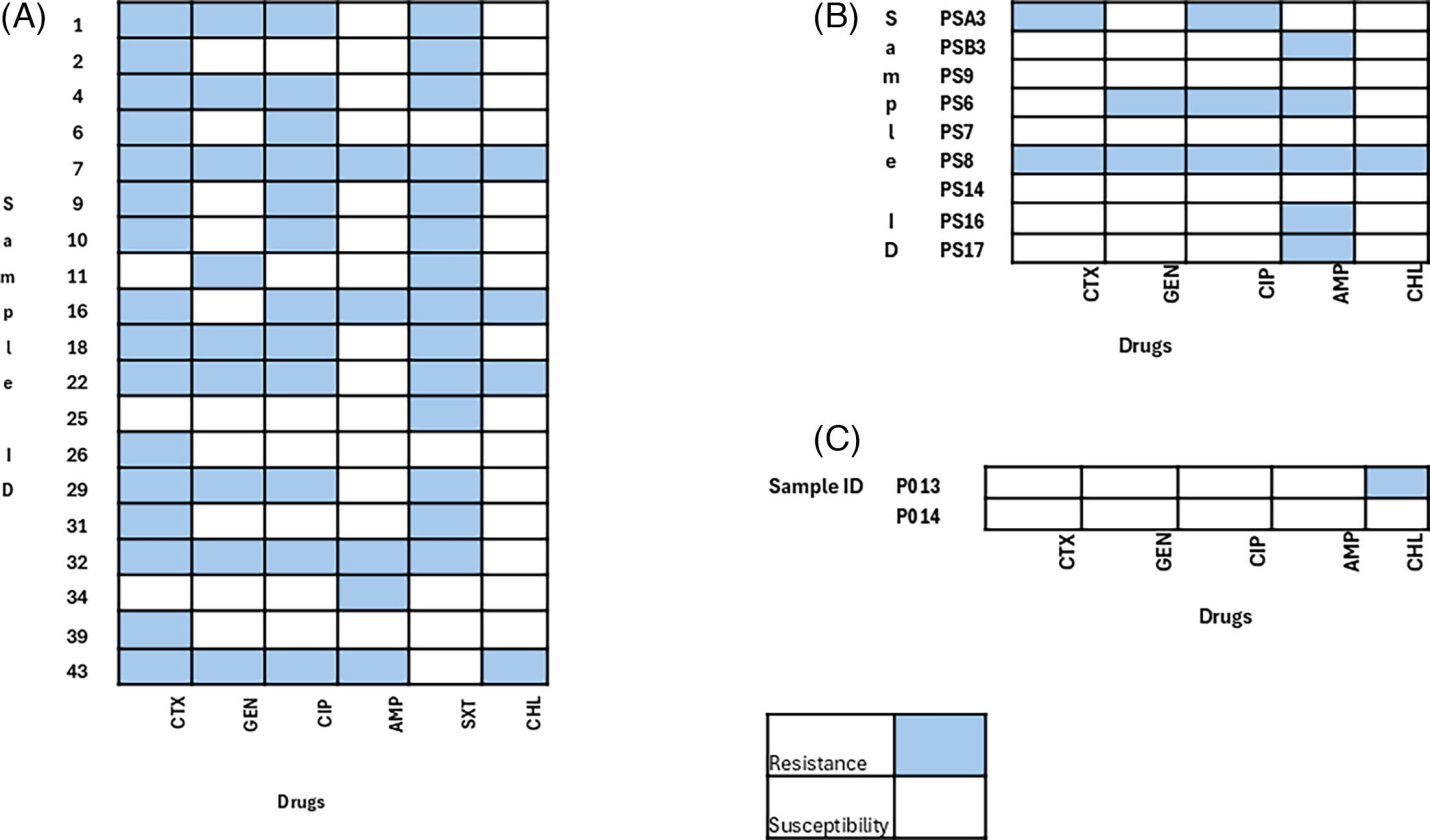
MDR patterns for *E. coli* in (A) fresh, (B) sun-dried, and (C) oven-dried samples.

Of the 19 *E. coli* isolates from fresh BSFL, 11/19 (58%) were resistant to multiple antibiotic classes commonly used, such as cefotaxime, gentamicin, chloramphenicol, ciprofloxacin, ampicillin, and co-trimoxazole. Meanwhile, of the nine *E. coli* isolates from sun-dried BSFL samples, only two isolates (PS6, PS8) showed resistance to three or more antibiotic classes, representing 22% (2/9). However, none of the isolates from the two oven-dried samples showed multi-drug resistance (MDR) (Fig. 4). An isolate was classified as multi-drug resistant if it exhibited resistance to at least three different antimicrobial courses**,** as defined by Magiorakos et al. (14).

### Multi-drug resistance in *Staphylococcus* isolates

**Fig 5:**
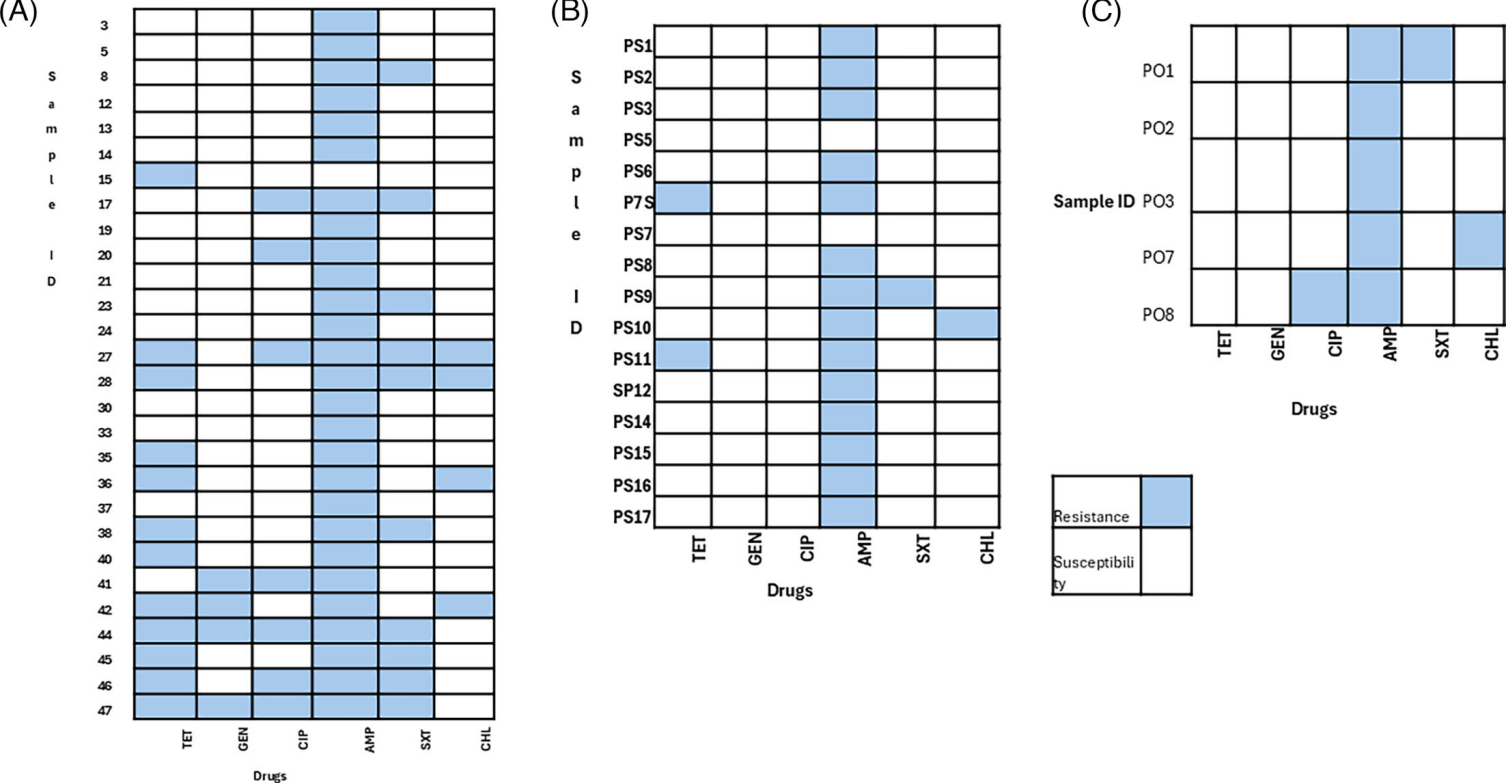
MDR results for *Staphylococcus* based on CLSI guidelines: (A) fresh samples, (B) sun-dried samples, and (C) oven-dried samples,

Of the 28 *Staphylococcus* spp. isolates from fresh BSFL samples, 11 showed MDR to 3 or more of the antibiotic classes, representing 39.3%, while none of the isolates from the sun-dried and oven-dried samples showed multidrug resistance (Fig. 5).

### Detection of AMR genes

**Fig 6:**
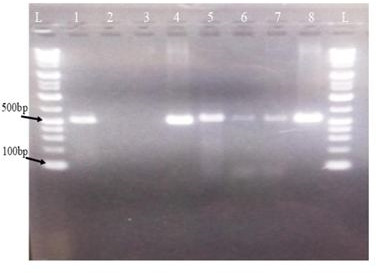
Lane L is DNA marker, lane 1 is positive *bla*_TEM_ (516 bp) gene, lanes 2 and 3 are negative, lanes 4 and 8 are positive controls, and lanes 5, 6, and 7 (7 from processed samples) are *bla*_CTX-M_ (544 bp) genes.

PCR was used to screen *E. coli* isolates for genes associated with extended-spectrum β-lactamase (ESBL), and bands of the expected size were regarded as positive results. Despite the high MDR rate in fresh samples, only 3 out of 19 (3/19; 16%) *E. coli* isolates tested positive for the ESBL genes. Two of which tested *bla*_CTX-M_ only, and one tested both *bla*_CTX-M_ and *bla*_TEM_ genes. The *bla*_CTX-M_ and *bla*_TEM_ genes were detected with band sizes of 544 bp and 516 bp, respectively, as shown in Fig. 6. From the processed BSFL *E. coli* isolates, only one (1/9, 11%) isolate from the sun-dried sample tested positive for the *bla*_CTX-M_ gene, which yielded a 544 bp band. However, none of the strains tested positive for *bla*_SHV_ or *bla*_OXA_.

Meanwhile, the *mecA* gene in *Staphylococcus* spp. associated with resistance to anti-*Staphylococcus* penicillins such as methicillin and cloxacillin was not detected. Phenotypically, the *Staphylococcus* strains from both fresh and processed samples exhibited cloxacillin resistance at 94% (47/50)

## DISCUSSION

In this study, we compared processed BSFL using two traditional methods and compared their effectiveness at reducing the bacterial load. From a total of 131 isolates, the majority of isolates were from the fresh BSFL (79/131; 60%) as compared to isolates from the sun-dried (37/131; 28%) and oven-processed (15/131; 12%) BSFL samples. The observed differences are likely due to the effects of sample processing that could have killed some of the bacterial cells in the dried BSFL. While various genera were identified, we focused on *E. coli* and *Staphylococcus* spp. as gram-negative and gram-positive models, respectively, for AST and AMR gene detection, as they are of public health significance. In some cases, *E. coli* harbor ESBLs that hydrolyze β-lactam antibiotics like cephalosporins and penicillin, leading to resistance (22). In addition, some *E. coli* commensals can transfer AMR genes to pathogenic bacteria by horizontal gene transfer using mobile genetic elements like plasmids, transposons, and insertion sequences (23), resulting in poor treatment outcomes.

Similarly, the genus *Staphylococcus* was selected since some strains are potentially pathogenic and contain the *mecA* gene that encodes resistance to β-lactam antibiotics (19). A typical example is methicillin-resistant *Staphylococcus aureus* (MRSA), a zoonotic pathogen that causes severe infections in humans, including pneumonia, endocarditis, skin and soft tissue infections, and mastitis and dermatitis in farm animals that can be difficult to treat (24).

The average bacterial load in fresh BSFL was 2.9 × 10⁷ CFU/mL, showing that unprocessed larvae possess high amounts of bacteria, as has been observed by other workers (9). Notably, gram-positive bacteria were more diverse (59/79; 75%) than gram-negative isolates (20/79; 25%) in fresh BSFL samples. This is probably because BSFL contain antimicrobial peptides (AMPs) that mainly inhibit the growth and proliferation of gram-negative bacteria (25). To a lesser extent, even the growth of gram-positive species can be inhibited by BSFL AMPs, especially *S. aureus,* including MRSA (26, 27).

The processed BSFL bacterial load revealed that both oven-drying and sun-drying suppressed bacteria to levels below the acceptable limit (10^9^ CFU/mL) (28) for insects to be safely used as animal feed. While the results highlight that both methods effectively ensure food safety, oven-drying was the most significant. This was probably due to oven-heating compared to sun-drying, whose temperature depended on the day’s weather. Furthermore, sun-drying was done in the month of May when the temperatures are usually low in Zambia, with a mean average temperature of 19.9°C (29). Moreover, a previous study by Nyangena et al. (9) reported a similar effect at 60°C, although their CFU decrease was smaller than in the present study. Similar to Nyangena et al. (9), our findings suggest that a higher temperature is more effective at reducing bacterial loads. However, the temperature should not be raised above a certain threshold if the nutritional value of the larvae is to be maintained (30). Therefore, an optimum temperature should be identified to balance the safety and maintenance of nutrients. While the methods used in this study achieved the desired outcome, some bacterial growth was still observed in the oven-dried samples. Nevertheless, we did not screen these isolates for heat resistance genes; thus, future studies should explore this area further.

*E. coli* are among the most common pathogens in hospital and community bacterial infections (31). Therefore, AMR in *E. coli* threatens public health, leading to adverse health outcomes among patients. In this study, some *E. coli* isolated from BSFL were resistant to commonly used antibiotics such as ampicillin, chloramphenicol, ciprofloxacin, co-trimoxazole, and gentamicin. In addition, some of the isolates possessed ESBL genes. Since the BSFL were collected from a dumpsite exclusively composed of poultry wastes, we speculate that the ESBL producers could have come from poultry. In Zambia, ESBL-producing *E. coli* have been reported in cloacal swabs obtained from chickens (32), probably because antibiotics are frequently used for growth promotion in the poultry industry (33). Furthermore, the clonal transmission of ESBL producers between poultry and humans was recently reported in Zambia (34). However, the strains presented in this study will need further characterization to determine their relationship with poultry and human strains previously reported.

In processed BSFL, less MDR was observed in *E. coli*, with only two isolates of the sun-dried samples (2/9; 22%) showing MDR compared to the 58% (11/19) MDR observed in the fresh BSFL samples. This could imply that processing kills most bacteria, including those exhibiting MDR. This indicates that there is very little chance of spreading multidrug-resistant strains that are difficult to treat through processed BSFL. Moreover, only one isolate (1/9; 11%) tested positive for the *bla*_CTX-M_ gene among the processed samples, compared to the fresh BSFL, which had three (3/19; 16%) positives (two *bla*_CTX-M_ and one with *bla*_TEM_). The results further highlight the limitations of our targeted genotypic characterization approach. While *bla*_CTX-M_ and *bla*_TEM_ are the commonest β-lactamase genes in Zambian clinical and poultry strains (35), the absence of these two genes could suggest the presence of other ESBL types. Therefore, there is a need to characterize the current *E. coli* strains using robust methods like whole-genome sequencing.

Gram-positive bacteria isolated from BSFL were more diverse than gram-negative bacteria isolated from the same sample. This observation could be explained by the inhibitory effect of antimicrobial peptides, which is more pronounced in gram-negative species. Despite the predominance of *Staphylococcus* spp. in this study, none of the isolates tested positive for the *mecA* gene, despite some isolates exhibiting resistance phenotypically. This observation could emanate from possible differences in the strains of *Staphylococcus aureus* (26, 27).

The public health implications of ESBL producers in antimicrobial resistance cannot be overemphasized as they present an emerging global threat to human, animal, and environmental health (36, 37). The presence of AMR and ESBL genes in BSFL is of great public health concern, given the widespread use of BSFL as feed in several communities, especially in low-income countries (38). Since the BSFL samples in this study were collected from the “wild” (poultry farm), the presence of ESBL producers and other AMR bacteria could indicate their widespread environmental existence. This has the potential risk of creating an avalanche of resistance in the environment (36) and can lead to the emergence of new and novel zoonotic diseases (37), if adequate care in use, management, and surveillance of the antibiotics in livestock and the environment is not taken (39). Increased use of antibiotics in poultry and domestic animals has the potential to increase the concentrations of these chemicals in the environment, with a concomitant increase in the proliferation and transmission of antibiotic-resistant bacteria (36). As observed elsewhere (40), an increase in the relative numbers of AMR bacteria and antibiotic resistance genes can partly be related to the environmental antibiotic selection pressure from enteric bacteria emanating from livestock.

### Conclusion

In this study, we elucidated the bacterial composition of BSFL obtained from a poultry dumpsite in Zambia’s Kitwe district. We found various bacterial genera, with a higher diversity among gram-positive isolates. We also compared the effectiveness of two traditional processing methods for BSFL, sun-drying and oven-drying, and found that both methods significantly reduced the bacterial load, with oven-drying causing a larger reduction. Molecular characterization of the isolated strains revealed *bla*_CTX-M_ and *bla*_TEM_ genes among *E. coli*, but the *mecA* gene was not detected among *Staphylococcus*. Altogether, the study revealed that BSFL harbor bacteria of zoonotic significance but can be reduced to acceptable levels with good processing methods, and thus can be considered as an animal feed alternative.
